# Targeting of 3D oral cancer spheroids by αVβ6 integrin using near-infrared peptide-conjugated IRDye 680

**DOI:** 10.1186/s12935-024-03417-y

**Published:** 2024-06-29

**Authors:** L. Dirheimer, T. Pons, A. François, L. Lamy, S. Cortese, F. Marchal, L. Bezdetnaya

**Affiliations:** 1 Centre de Recherche en Automatique de Nancy, Centre National de la Recherche Scientifique, UMR 7039, Université de Lorraine, Vandoeuvre-lès-Nancy, France; 2 ESPCI Paris, LPEM UMR 8213, PSL University, CNRS, Sorbonne University, Paris, France; 3 Research Department, Institut de Cancérologie de Lorraine, 6 avenue de Bourgogne, Vandoeuvre-lès-Nancy, 54519 France; 4 Surgical Department, Institut de Cancérologie de Lorraine, 6 avenue de Bourgogne, Vandoeuvre-lès-Nancy, 54519 France

**Keywords:** Head and neck cancer, Oral cancer, Tongue cancer, αVβ6 integrin, Spheroid, Fluorescence imaging, IRDye, Near Infrared fluorescence (NIR)

## Abstract

**Background:**

In the treatment of oral cavity cancer, margin status is one of the most critical prognostic factors. Positive margins are associated with higher local recurrence and lower survival rates. Therefore, the universal goal of oral surgical oncology is to achieve microscopically clear margins. Near-infrared fluorescence guided surgery (FGS) could improve surgical resection using fluorescent probes. αVβ6 integrin has shown great potential for cancer targeting due to its overexpression in oral cancers. Red fluorescent contrast agent IRDye 680 coupled with anti-αVβ6 peptide (IRDye-A20) represents an asset to improve FGS of oral cancer. This study investigates the potential of IRDye-A20 as a selective imaging agent in 3D three-dimensional tongue cancer cells.

**Methods:**

αVβ6 integrin expression was evaluated by RT-qPCR and Western Blotting in 2D HSC-3 human tongue cancer cells and MRC-5 human fibroblasts. Targeting ability of IRDye-A20 was studied in both cell lines by flow cytometry technique. 3D tumor spheroid models, homotypic (HSC-3) and stroma-enriched heterotypic (HSC-3/MRC-5) spheroids were produced by liquid overlay procedure and further characterized using (immuno)histological and fluorescence-based techniques. IRDye-A20 selectivity was evaluated in each type of spheroids and each cell population.

**Results:**

αVβ6 integrin was overexpressed in 2D HSC-3 cancer cells but not in MRC-5 fibroblasts and consistently, only HSC-3 were labelled with IRDye-A20. Round shaped spheroids with an average diameter of 400 μm were produced with a final ratio of 55%/45% between HSC-3 and MRC-5 cells, respectively. Immunofluorescence experiments demonstrated an uniform expression of αVβ6 integrin in homotypic spheroid, while its expression was restricted to cancer cells only in heterotypic spheroid. In stroma-enriched 3D model, Cytokeratin 19 and E-cadherin were expressed only by cancer cells while vimentin and fibronectin were expressed by fibroblasts. Using flow cytometry, we demonstrated that IRDye-A20 labeled the whole homotypic spheroid, while in the heterotypic model all cancer cells were highly fluorescent, with a negligible fluorescence in fibroblasts.

**Conclusions:**

The present study demonstrated an efficient selective targeting of A20FMDV2-conjugated IRDye 680 in 3D tongue cancer cells stroma-enriched spheroids. Thus, IRDye-A20 could be a promising candidate for the future development of the fluorescence-guided surgery of oral cancers.

**Graphical Abstract:**

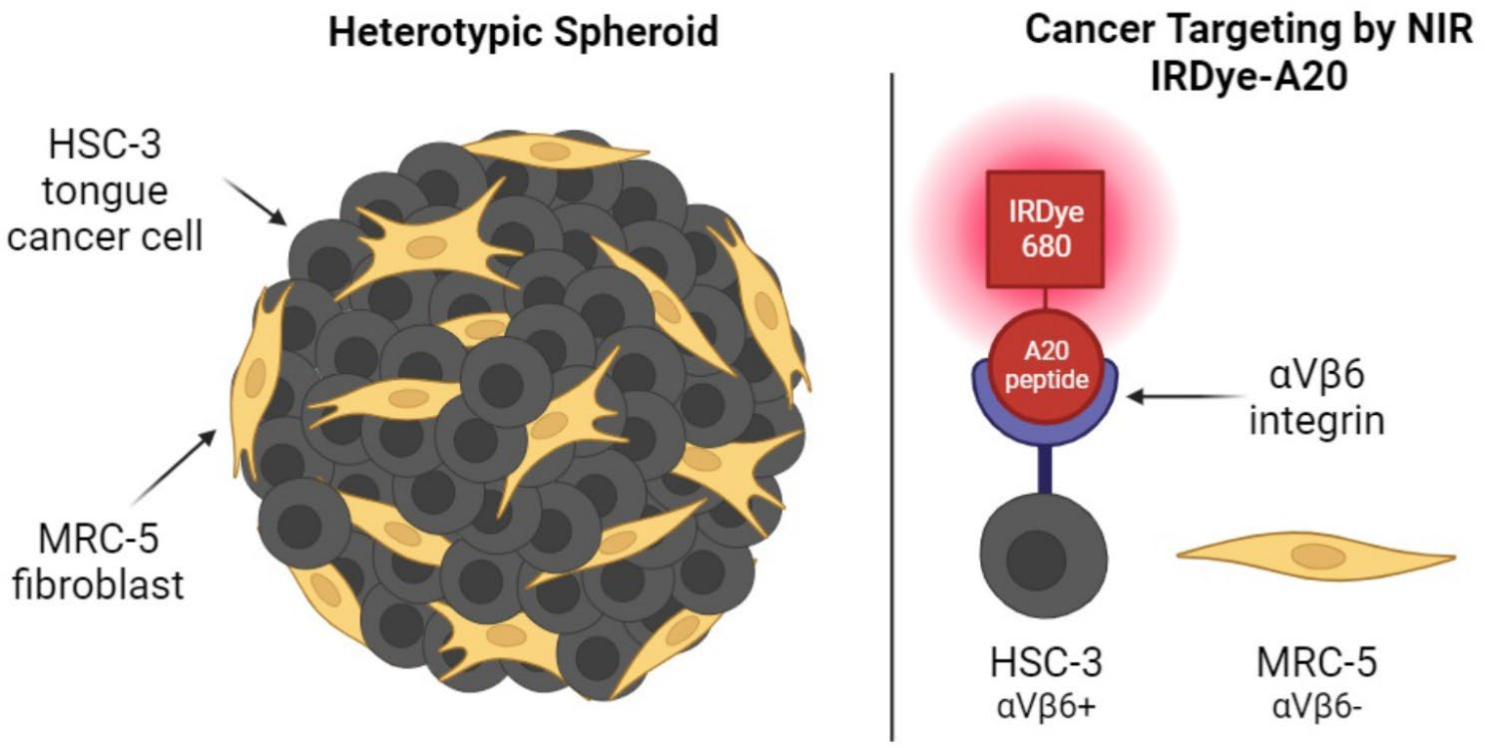

**Supplementary Information:**

The online version contains supplementary material available at 10.1186/s12935-024-03417-y.

## Background

More than 350,000 new oral cancer cases are diagnosed annually worldwide [1]. Over 90% f these cases are oral squamous cell carcinoma (OSCC) with a mortality rate reaching over 60% in five years [2]. Within the oral cavity, tongue appears as the main cancer subsite with at least 40% prealence [3–6]. Surgical resection is a primary choice of treatment of oral cancer and achieving microscopically clear margins is the primary goal of head and neck surgical oncology. This issue is of utmost importance in tongue cancers since the tongue plays a vital role in physiological functions such as speech, swallowing, and chewing [7, 8]. Therefore, the glossectomy-induced tongue defects can seriously affect patients’ quality of life (QoL). From this point of view, a novel technique such as fluorescence–guided surgery (FGS) aiming to ensure a safe margin during surgery could be a more delicate option for tongue cancer patients. FGS is an intraoperative technique, which relies on fluorescent contrast agents to highlight cancerous tissue and microlesions. Untargeted fluorescent tracers such as red light emitting 5-ALA-PpIX [9], or a class of infrared emitting dyes like Indocyanine Green (ICG) [10, 11] appeared to be effective agents for in vivo and ex vivo delineation of surgical margins in oral oncology. In recent years, the field has shifted from using untargeted fluorescent tracers to the tracers directed towards tumor-specific characteristics in order to overcome its nonspecific uptake [12, 13].

Among molecular targets for FGS, the epidermal growth factor receptor (EGFR) appears as the most studied biomarker as it is overexpressed in 90% of OSCC [14]. Several studies have demonstrated that anti-EGFR antibodies such as Cetuximab and Panitumumab, coupled with near infrared (NIR) fluorescent dyes IRDye 800CW, allowed a better identification of tumor margins during FGS and improved an *ex-vivo* analysis of specimens [12, 13, 15, 16].

Another interesting target for FGS could be integrins. Integrins are heterodimeric transmembrane glycoproteins composed of two subunits, α and β, which bind to form 24 isoforms, divided into 4 families depending on their ligands [17, 18]. They are involved in intercellular and cell-extracellular matrix interactions, and thus play numerous roles in cell biology and cancer development by modulating proliferation, cell survival, migration and angiogenesis [19]. Among the family of integrins, the αVβ6 integrin is the most attractive target for two reasons. It is expressed in most oral cancers and especially at the invasive margins [20, 21]. Secondly, compared to EGFR, αVβ6 is not or only poorly expressed in normal tissue [22], thus improving the contrast between healthy and tumor tissues. This integrin has been shown to be involved in cell adhesion, migration and invasion, proliferation and survival, promotion of tumor growth in mice [21, 23] and is also able to induce the formation of pro-tumoral stroma [24, 25].

Specific targeting of the αVβ6 integrin can be achieved through A20FMDV2 (A20) peptide, a 20-mer peptide (NAVPNLRGDLQVLAQKVART) derived from the foot-and-mouth disease virus. This peptide has high affinity towards αVβ6 integrin [26, 27] with an inhibiting ligand binding IC_50_ of 3 nM [28]. A20 peptide has found several applications such as drug delivery, imaging, oncoviruses therapy and chimeric antigen receptor T-cells (CAR T-cells) therapy [29].

Several studies have been carried out on A20FMDV2 conjugates in preclinical models for in vivo PET imaging of pancreatic cancers [28, 30] demonstrating its selective binding to αVβ6 positive tumors. Fluorescence imaging of A20 conjugated peptide was performed in the work of Ganguly et al., where A20 peptide was bipegylated and coupled with IRDye 800CW (IRDye800-PEG_28_-A20FMDV2-K16R-PEG_28_) [31]. This probe, used on xenografted pancreatic tumor bearing mice, was efficiently accumulated in αVβ6 + tumors. The use of A20-peptide conjugated with IRDyes for surgical margin assessment in HNSCC is poorly explored.

It has gradually become clear that compared to 2D monolayers, 3D culture systems can better mimic native tumor microenvironment, bridging a gap between 2D monolayers and animal models, and even may be able to replace them [32, 33]. In order to better assess a complex cellular interaction within the tumor microenvironment, stroma–rich spheroids have been proposed in numerous studies for head and neck cancers [34, 35]. Stromal cells and in particularly fibroblasts are prominent in solid tumor microenvironment and contribute to the remodeling of extracellular matrix (ECM), to the enhancement of pro-tumor immune response and are involved in drug resistance [36, 37].

In the present work, we established and characterized a 3D spheroid model of human tongue squamous cell carcinoma enriched with human cultured fibroblasts to better recapitulate tumor microenvironment. Spheroid cancer cells overexpressing αVβ6 integrin were targeted using the A20FMDV2 peptide coupled with IRDye 680CW (IRDye-A20). Targeting of both homo- and hetero-typic spheroids with IRDye-A20 was studied using flow cytometry and fluorescence microscopy to assess its potential as a selective imaging agent.

## Methods

### Cell lines

The human tongue squamous cell carcinoma cell line HSC-3 (SCC193) was purchased from Sigma-Aldrich (USA) and cultured in Dulbecco’s Modified Eagle Medium (DMEM, Gibco, United Kingdom) supplemented with 9% heat-inactivated fetal bovine serum (FBS, Sigma-Aldrich, France), sodium pyruvate 1.25 mM (Gibco, China), essentials amino acids 0.5X, nonessentials amino acids 0.5X, vitamins 0.4X (all from Gibco, United Kingdom), L-glutamine 2 mM and L-serine 10.8 ng/mL (Sigma-Aldrich, United Kingdom), L-asparagine 20 ng/mL (Fisher, Japan). The human embryonic lung fibroblast cell line MRC-5 (Cat. No: CCL-171, USA) and MeWo fibroblasts (Cat. No: HTB-65TM), derived from human melanoma, were purchased from ATCC and cultured in Minimum Essential Medium (MEM, Sigma Aldrich, France) supplemented with 9% heat-inactivated FBS and sodium pyruvate 1 mM. The hypopharynx cancer cells FaDu (Cat. No: HTB-43) and adenocarcinoma cell line HT-29 (Cat. No: HTB-38) were purchased from ATCC and cultured in Roswell Park Memorial Institute 1640 medium (RPMI-1640, Invitrogen, USA) supplemented with 9% heat-inactivated FBS and 2 mM glutamine. Cells were maintained in incubator at 37 °C with 5% CO_2_ and reseeded every week to ensure exponential growth. All cells were negative for mycoplasma contamination.

### Spheroids formation

Multicellular tumor spheroids were generated using the liquid-overlay technique as described previously [38]. For homotypic HSC-3 spheroids (H10), HSC-3 cells (10^4^ cells in 200 µL) were added to each well of a 96-well plate pre-coated with 1% aarose (w/v in water) and incubated at 37 °C with 5% CO_2_. For HSC-3/MRC-5 heterotypic spheroids (H5M5), HSC-3 cells were pre-incubated overnight with 2 µg/mL of poly(lactic-co-glycolic acid) (PLGA) polymer nanoparticles pre-loaded with cyanine dyes (DiO) [39]. Afterwards, pre-stained HSC-3 cells (5 × 10^3^ in 100 µL) were seeded in 96-well plates. After 3 days, 100 µL of MRC-5 cells were added to each well (5 × 10^3^ cells/well), either unlabeled for flow cytometry analysis, or after overnight pre-incubation with PLGA polymer nanoparticles loaded with Dil for fluorescence microscopy. After 24 h, heterotypic spheroids were taken for analysis. Spheroids growth was monitored using inverted Olympus CK2 optical microscope (Olympus, France). PLGA polymer nanoparticles loaded with cyanine dyes DiO and Dil were kindly provided by Dr A Klymchenko (Université de Strasbourg, France).

### Spheroids characterization

Spheroids were embedded in tissue freezing medium (TFM, Microm Microtech, France), frozen at -80 °C, and cut into 10 μm thick sections using a cryostat (Leica CM 1850, Leica Biosystems, Germany). Cryosections were fixed in 4% paraformaldehyde solution for 10 min, followed by hematoxylin and eosin (HE) staining, or fixed with 96% ethanol for 10 min, followed by immunohistological analysis. KI-67 immunohistochemistry (IHC) was performed using anti-human KI-67 mouse monoclonal antibody (Dako Santa Clara, US) on BechmarkUltra IHC automate Roche (Roche diagnostics, Rotkreuz, Switzerland). For immunofluorescence studies (IF), cryosections were blocked with 3% bovine serum albumin in phosphate buffered saline solution (PBS) for 1 h at room temperature. Cryosections were incubated overnight at 4 °C with either rabbit anti-αVβ6 (Biorbyt, 100,289, 1:100), rabbit anti-Fibronectin (Abcam, 2413, 1:100), rabbit anti-Vimentin (Cell Signaling Technology, 5741, 1:100), mouse anti-cytokeratin 19 (Santa Cruz, 6278, 1:100), mouse anti-E-cadherin (BD Bioscience, 610,181, 1:100) antibodies, followed by secondary goat anti-rabbit Atto 633 antibody (Sigma, 41,176, 1:400) or secondary goat anti-mouse Atto 633 antibody (Sigma, 78,102, 1:400) for 1 h at room temperature. After nuclear counterstaining with DAPI (Vectashield with DAPI, Vector laboratories, USA), cryosections were observed under epifluorescence microscope (Olympus AX-70, Olympus, France) equipped with a CoolLED pe-4000 system (CoolLed, UK). Fluorescence from DAPI was detected using a 420–460 nm filter (λexc = 365 nm) and signal from Atto 633 conjugated antibody with a 652–682 nm filter (λexc = 635 nm) was registered. Quantification of fluorescence intensity was performed with ImageJ software using the following formula: Mean Fluorescence Intensity (MFI) = Integrated density – (Area of spheroids x mean background fluorescence). DiO fluorescence was detected with a 510–550 nm filter (λexc = 460 nm) and that of Dil with a 560–600 nm filter (λexc = 550 nm).

### Proteomic expression of ITGB6 in normal tissue and primary HNSCC tumor

ITGB6 (β6 integrin subunit) proteomic expression profile in normal tissue and primary HNSCC tumor was obtained using UALCAN (The University of ALabama at Birmingham CANcer data analysis Portal, https://ualcan.path.uab.edu), a web resource for analyzing cancer OMICS data [40, 41], using data from Clinical Proteomic Tumor Analysis Consortium (CPTAC).

### Quantitative reverse-transcription PCR

Total RNA was extracted using RNeasy Mini Kit (Qiagen, Hilden, Germany) and quantified using Invitrogen™ Qubit RNA HS Assay Kit (ThermoFisher Scientific, Inc. Waltham, MA, USA) according to manufacturer’s instructions. cDNA were synthetized using iScript™ cDNA Synthesis Kit (Bio-Rad, Hercules, CA, USA) with 1 µg of total RNA. qPCR was performed on LighCycler^®^480 (Roche, Basel, Switzerland) using LightCycler^®^ 480 SYBR Green I Master kit. Primers’ sequences, purchased from Eurogentec (Seraing, Belgium) are described in Table 1. Results were normalized using β-Actin and gene expression was determined by Livak method (2^−ΔΔCt^), using (αVβ6+) cell line HT-29 as reference [42].

**Table 1:** RT-qPCR primer sequences

Gene	Forward Sequence	Reverse Sequence	REF
ITGB6	GCAAGCTGCTGTGTGTAAGGAA	CTTGGGTTACAGCGAAGATCAA	[43]
ACTB	AGAGCTACGAGCTGCCTGAC	AGCACTGTGTTGGCGTACAG	[44]

### Western blotting

Cell pellet was lysed with ice-cold 1X RIPA lysis buffer (Merck Millipore, USA) supplemented with 2 mM phenylmethylsulfonyl fluoride (PMSF, Sigma, USA). Protein concentration was quantified using DCTM Protein Assay Kit (Bio-Rad). 40 µg of protein lysate was loaded to 7.5% non-reducing SDS PAGE and after 150 min of migration and 90 min of blotting, PVDF membrane was saturated with 5% (w/v) solution of non-fat powered milk in TBST (Tris buffer solution with 0.1% Tween-20) for 1 h. Membrane was incubated overnight at 4 °C with either 1:100 mouse anti-human β6 integrin subunit antibody (Merck Millipore corp, USA, 407,317) or 1:1000 mouse anti-α-tubulin antibody (Santa Cruz Biotechnology, 23,948), followed by incubation with 1:2000 anti-mouse IgG HRP-conjugated secondary antibody (Cell signaling technology, 7076 S) for 1 h at room temperature. Proteins were detected by chemiluminescence using the Clarity Western ECL kit (Bio-Rad) in gel imager (Azure C600, ScienceTec, USA). Band density, normalized to that of tubulin was quantified using ImageJ software (NIH, USA).

### Peptides-IRDye 680 conjugate synthesis

IRDye 680LT was purchased from LI-COR Biosciences (USA). The peptides were synthesized at the Plateforme d’Ingénierie des Protéines (Sorbonne Université, Paris, France) by solid-phase synthesis followed by HPLC purification. The A20 peptide is based on the A20FMDV2 sequence, with a flexible glycine-serine linker and N-terminal cysteine, with the following sequence: CGGGSGGGSVPNLRGDLQVLAQKVART. The control scrambled sequence has the following sequence: CGGGSGGGSLRDQTGLKNPVQLARVAV [45, 46]. The peptides were reacted overnight at room temperature for 12 h with the maleimide dyes in dimethylsulfoxide (50 mg/mL peptide, 1:2 dye: peptide ratio) and purified by precipitation with acetone and dried. The structural formula of IRDye-A20 is shown in Supplementary Fig. S1. Peptide conjugation had little impact on IRDye 680 optical properties. Indeed, max absorbance peaked at 680 nm for both IRDye, IRDye-A20 and IRD-Scr, while an increase of fluorescence by 25% was observed for IRDye-A20 and IRDye-Scr compared with IRDye alone in medium supplemented with 9% FBS (Supplementary Fig. S2).

### Incubation of 2D and 3D cells with IRDye-conjugates

For 2D monolayers, cells were seeded in P24 well plates using 500 µL of HSC-3 (4.10^4^ cells/mL) or MRC-5 (5.10^4^ cells/mL) cell suspensions. After 5 days, culture medium was removed and cells were washed with PBS prior to incubation with IRDye-conjugates diluted in serum-free medium. For endocytosis blockade, incubation with IRDye-conjugate was carried out either at 37 °C or at 4°C. For inhibition binding assay, HSC-3 cells were co-incubated for 3 h with IRDye-A20 (50 nM) and increasing concentrations of free unlabeled A20FMDVM2 peptide. For specific endocytosis inhibition, HSC-3 cells were pre-incubated with either Genistein (400 µM), Chlorpromazin (50 mg/mL) or (5-(N-Ethyl-N-isopropyl)-Amiloride) (EIPA, 100 µM) for 30 min. Afterwards, 500 nM of IRDye-A20 was added and further incubated for 3 h. For 3D spheroids, half of the spheroid culture medium was removed and further placed in incubator (37 °C, 5% CO_2_) with a double concentrated IRDye conjugate solution for 1–24 h.

### Flow cytometry

Cell monolayers were washed twice with PBS before subsequent trypsinization. Spheroids were washed in PBS and trypsinized with half-diluted trypsin during 25 min under agitation in incubator at 37 °C. Trypsinization was stopped by addition of complete medium and spheroid dissociation was completed mechanically with pipette. In both cases, cell suspension was centrifuged (300 g, 5 min) and resuspended in serum-free medium. Flow cytometry analysis was performed using Accuri C6 Plus cytometer (BD, USA) equipped with 488 and 640 nm emitting lasers. After excitation at 488 nm, DiO fluorescence was detected with a 533 ± 30 nm filter in the fluorescence channel FL1 to discriminate HSC-3 (DiO +) and MRC-5 (DiO -) cells. IRDye fluorescence was detected with a 670 nm long pass filter in the fluorescence channel FL3 after excitation at 640 nm. For cocultured spheroids, HSC-3 and MRC-5 cells were discriminated using DiO staining and after that IRDye-conjugates uptake was evaluated separately in each cell population. Data analysis was performed with Accuri C6 Plus software (BD, USA).

### IRDye-A20 penetration in spheroids

H10 and H5M5 spheroids were incubated with 250 nM of IRDye-A20 or IRDye-Scr for 24 h. Afterwards, they were washed with PBS, frozen in TFM, cut in 10 μm thick sections and fixed with 96% ethanol for 10 min. IRDye 680 signal was observed under epifluorescence microscope with a 652–682 nm filter (λexc = 635 nm). Penetration profiles of IRDye-A20 in spheroids were obtained using ImageJ software. Briefly, 100 concentric circles were drawn in spheroids and the MFI was measured for each circle. The first circle (1) represents the spheroids periphery while the last one (100) represents its core. D_50_, the distance in spheroids matching 50% of maximum MFI, was computed from distribution profile by calculating the cumulative MFI and then relating 50% of its maximum to spheroids diameter.

### Crystal violet assay

Cells were seeded into 96-well plates (100 µL, 4.10^4^ cells/mL) for 5 days, then culture medium was replaced with 0.1–4 µM of IRDye-conjugates diluted in serum-free medium for 24 h at 37 °C. Afterwards, cells were washed, fixed with 70% ethanol for 10 min and further incubated with 0.2% crystal violet in 20% ethanol for 15 min. After several washes, crystal violet was solubilized using 0.1% acetic acid and 50% ethanol and optical density was recorded at 540 nm with Multiskan microplate readers (Ascent, Labsystems). Cell viability was calculated by normalizing to that of the control condition.

### Statistical analysis

Statistical analysis was performed using GraphPad Prism 9 (Boston, Massachusetts USA) and OriginPro 2021 (OriginLab Corporation, Northampton, MA, USA) software. Data are presented as mean ± SEM. Comparison of groups for αVβ6 RT-qPCR and Western-Blotting assays was carried out by the one-way ANOVA test followed by Tukey’s assay; IRDye-A20 accumulation in HSC-3 and MRC-5 cell monolayers and in H10 spheroids was analyzed by the two sample t test with Welch correction. The two sample t test was used for comparison of groups analyzed for β6 protein expression, for quantification of cell subtypes in H5M5 spheroid, for immunofluorescence quantification in H10 and H5M5 spheroids and D_50_ analysis in H10 and H5M5 spheroids. The One sample t test was used for inhibition binding assay. One-way ANOVA followed by Bonferroni assay was used for active transport inhibition. One-way ANOVA with Welch correction followed by Dunnett’s T3 assay was applied for IRDye-A20 accumulation in H5M5 spheroids. One-way ANOVA followed by Dunnett’s test was applied for endocytosis inhibition assay. Statistical significance was set at a p-value of ≤ 0.05.

## Results

### IRDye-A20 targeting of 2D cells

#### Analysis of αVβ6 expression

**Fig. 1:**
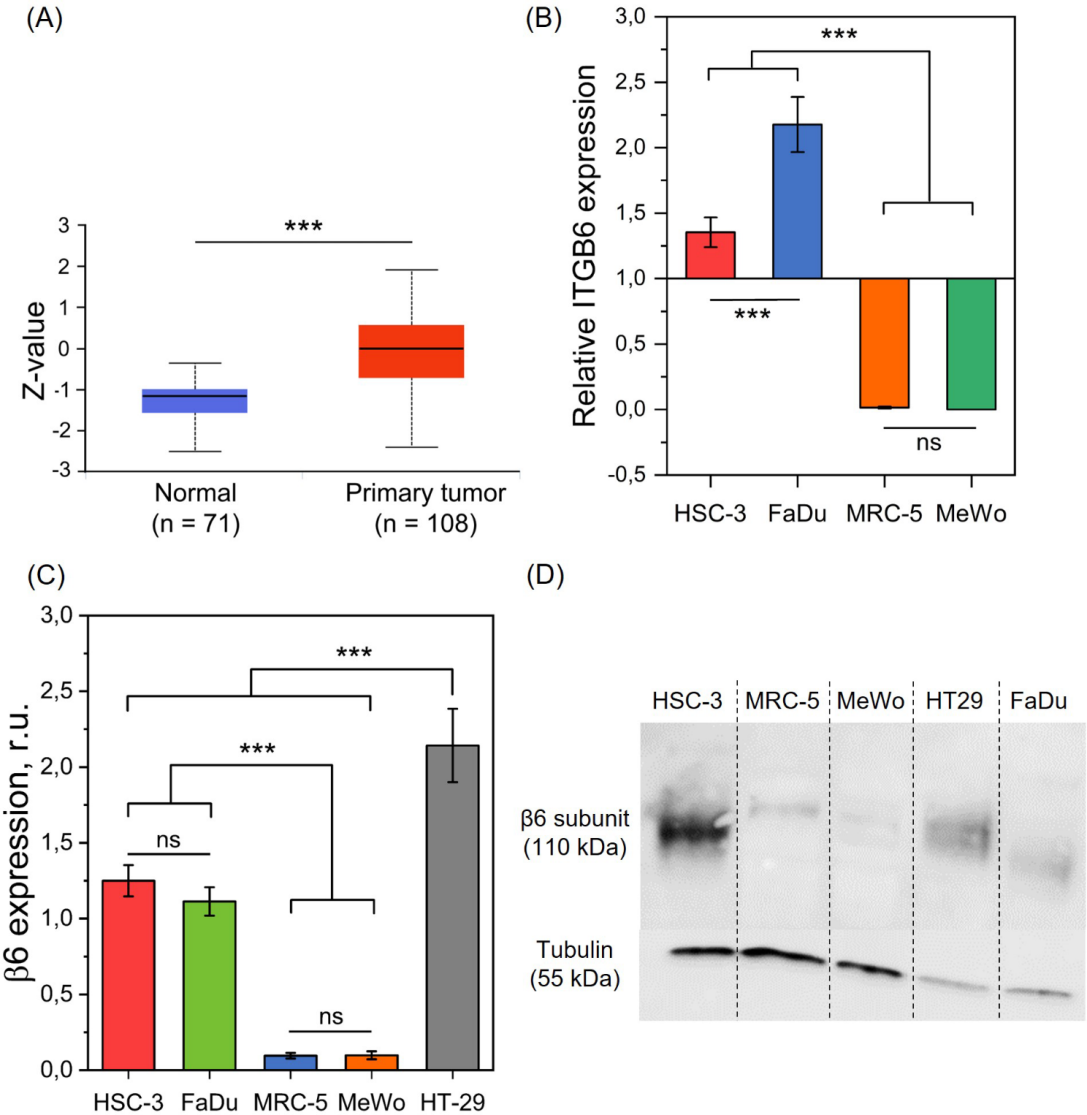
Analysis of αVβ6 expression. (**A**) ITGB6 proteomic expression profile in normal tissue and primary tumor (data from UALCAN) (*n* = 71–108; *** *p* < 0.001; Two sample t test). (**B**) RT-qPCR analysis of *ITGB6* mRNA expression; β-actin was used as housekeeping gene. The (αVβ6+) cell line HT-29 was used as reference (*n* = 3–8; *** *p* < 0.001; One-way ANOVA followed by Tukey’s assay). (**C**-**D**) β6 integrin subunit protein expression assessed by Western-Blot; tubulin was used as a loading control (*n* = 3–7; *** *p* < 0.001; One way ANOVA followed by Tukey’s assay). Data are presented as mean ± SEM

The ITGB6 (β6 integrin subunit) proteomic expression profile was analyzed in 71 normal tissues and 108 head and neck squamous cell carcinoma (HNSCC) primary tumors from CPTAC data, using the UALCAN data box-plot analysis portal (Fig. 1A). A significantly higher ITGB6 protein expression was found in primary HNSCC compared with normal tissues (*p* < 0.001) (Fig. 1A). This observation is consistent with another clinical report, where immunochemical staining of tumor tissue from HNSCC patients (*n* = 17) demonstrated a high tumor-border score for αVβ6 integrin [22]. Altogether, these data confirm the interest of this protein for selective active targeting.

Next, we evaluated the expression of αVβ6 integrin by both RT-qPCR and Western-Blot techniques in several HNSCC cells (HSC-3 and FaDu) and fibroblasts cells (MRC-5 and MeWo) (Fig. 1B-D). HSC-3 line was selected based on the report of Hsiao et al. [47], which demonstrated an overexpression of αVβ6 integrin. The HT-29 adenocarcinoma cell line served as a positive control for αVβ6 expression [42].

RT-qPCR results demonstrated that the relative *ITGB6* expression was significantly higher in HNSCC cells (HSC-3 & FaDu) than those in fibroblasts (MRC-5 & MeWo) (*p* < 0.001) (Fig. 1B). No difference in expression was evidenced between MRC-5 and MeWo cells (*p* > 0.05) while increased *ITGB6* expression was found in FaDu cells compared to HSC-3 (*p* < 0.001). Similarly, as assessed by Western-Blot (WB), the expression of β6 subunit (band at 120 kDa), which can only be associated with αV subunit, was 13-fold higher in HNSCC cells compared to fibroblasts (*p* < 0.001) (Fig. 1C-D). Similar protein expression was found between HSC-3 and FaDu cancer cells (*p* > 0.05), while β6 subunit was not expressed in MRC-5 and MeWo fibroblasts. This overexpression of β6 integrin in cancer cells assessed by Western Blotting is consistent with previous observations [47] where HSC-3 cancer cells demonstrated a high expression of β6 subunit compared to other cancer cell lines and myofibroblasts MeWo. Thus, both RT-qPCR and WB results were correlated, as the integrin was overexpressed only by cancer cells and not by fibroblasts.

### IRDye-A20 accumulation in 2D cells

**Fig. 2:**
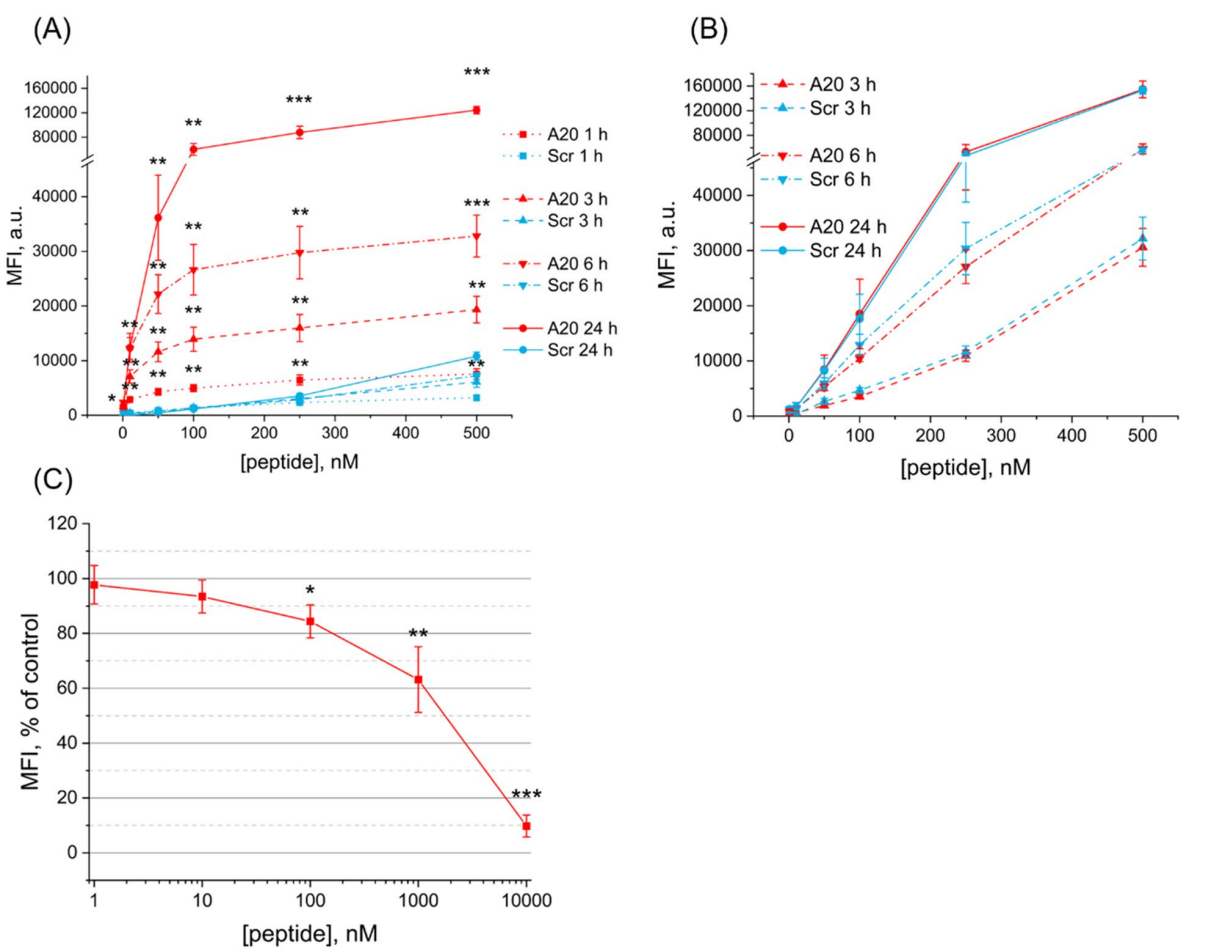
IRDye-A20 accumulation in cell monolayers assessed by flow cytometry analysis. Cell Mean Fluorescence Intensity (MFI) of HSC-3 (**A**) and MRC-5 (**B**) cells after incubation either with IRDye-A20 or IRDye-Scr in the concentrations range 1-500 nM, for 1 h, 3 h, 6 h and 24 h (*n* = 5–10; * *p* < 0.05; ** *p* < 0.01; *** *p* < 0.001; Two sample t test with Welch correction). (**C**) Inhibition binding assay. HSC-3 cells were co-incubated with IRDye-A20 (50 nM) and increasing concentrations of unlabeled A20 peptide for 3 h (*n* = 5–6; * *p* < 0.05; ** *p* < 0.01; *** *p* < 0.001; One sample t test). Data are presented as mean ± SEM

In the first place, accumulation of IRDye-A20 was assessed by flow cytometry in 2D monolayers in both tongue cancer cells HSC-3 and MRC-5 fibroblasts by measuring cell mean fluorescence intensity (MFI) (Fig. 2A-B). A scramble version of the A20 peptide (IRDye-Scr) was used as a control to capture the fraction of non-specifically delivered products. In HSC-3 monolayers, higher accumulation of IRDye-A20 compared to IRDye-Scr was observed at all incubation times and all peptide concentrations (Fig. 2A). The accumulation of IRDye-Scr was concentration-dependent, with non-specific binding that increased slightly at concentrations exceeding 250 nM. Different accumulation profile was characteristic for IRDye-A20 : a rapid accumulation occurred in the range 10–100 nM, after which MFI increased slightly until 500 nM. At 100 nM, MFI was 3.6-times higher after 1 h incubation with IRDye-A20 compared to scramble (4960 ± 650 a.u. vs. 1370 ± 230 a.u., *p* < 0.01). This difference in MFI values increased ca.50-fold after 24 h incubation (60 100 ± 9750 a.u. vs. 1160 ± 220 a.u., *p* < 0.01). To confirm specific A20 binding, we further conducted experiments with (αVβ6-) MRC-5 fibroblasts (Fig. 2B). Similar accumulation of IRDye-A20 and IRDye-Scr was achieved (*p* > 0.05) in fibroblasts irrespective of peptide concentration, confirming IRDye-A20 accumulation in (αVβ6+) cancer cells but not in normal (αVβ6-) cells. Toxicity of IRDye-A20 and IRDye-Scr was assessed by crystal violet assay in HSC-3 and MRC-5 cells after 24 h incubation. Loss of viability was not detected even at the highest concentration (4 µM) of IRDye conjugated to the peptides (*p* > 0.05) (Supplementary Fig. S3).

We further performed the inhibition binding assay by co-incubating HSC-3 cells with IRDye-A20 in the presence of increasing concentrations of unlabeled A20 peptide (Fig. 2C). Increasing concentrations of A20 peptide from 1 nM to 10 µM reduced progressively MFI with a complete inhibition of IRDye-A20 accumulation at 10 µM. All these experiments clearly demonstrated a specific targeting of HSC-3 cells by IRDye-A20.

### Internalization studies

**Fig. 3:**
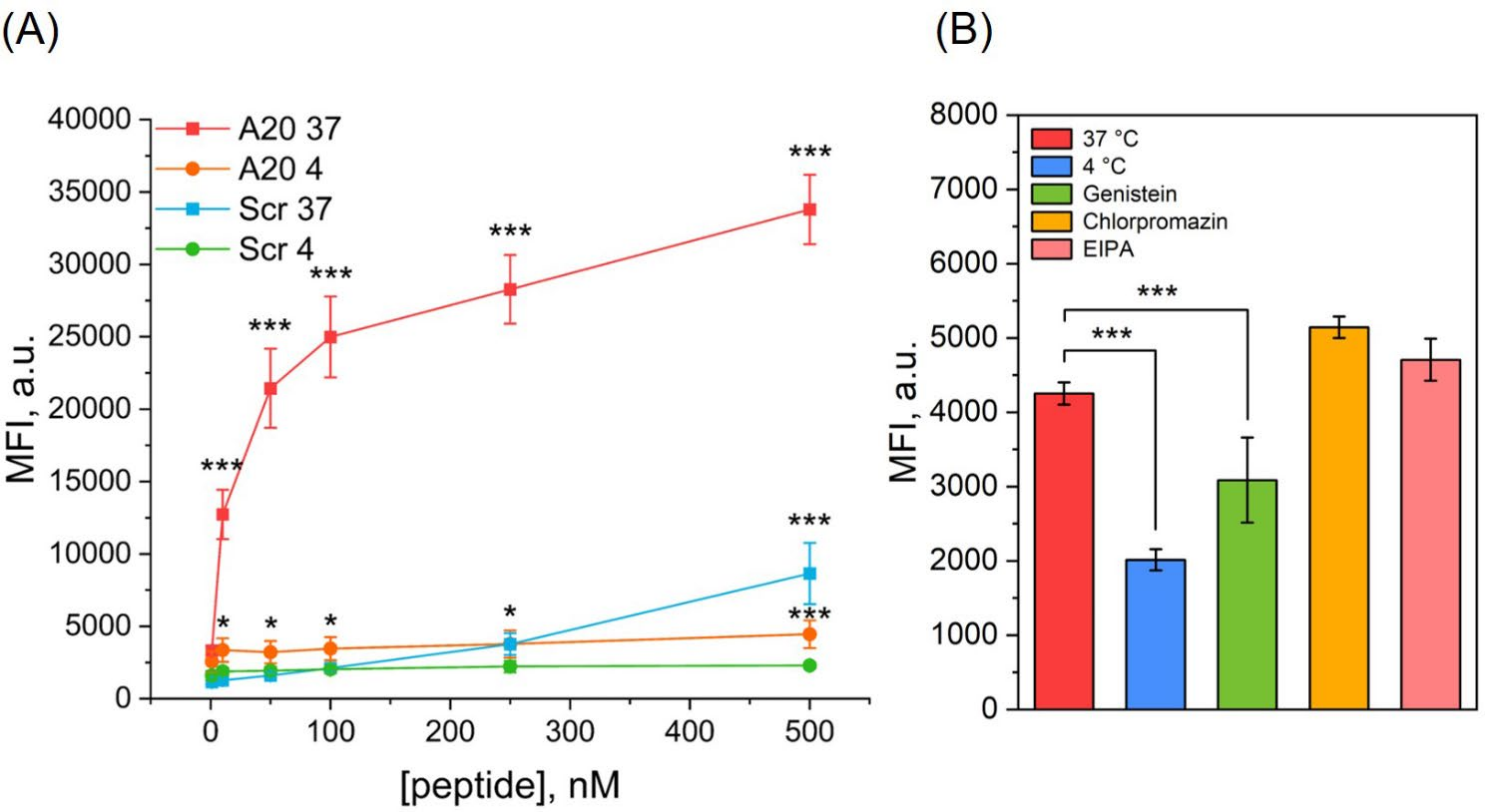
IRDye-A20 internalization studies in cell monolayers assessed by flow cytometry. (**A**) Active transport inhibition. HSC-3 cells were incubated with IRDye-A20 or IRDye-Scr in the concentration range 1-500 nM for 3 h at 4 °C or 37 °C, followed by flow cytometry analysis (*n* = 4; * *p* < 0.05; *** *p* < 0.001; One-way ANOVA followed by Bonferroni test). (**B**) Endocytosis inhibition. HSC-3 cells were pre-incubated 30 min with either genistein, chlorpromazine or EIPA followed by incubation with IRDye-A20 500 nM for 3 h, or directly incubated with 500 nM of IRDye-A20 for 3 h at 37 °C or 4 °C, and followed by flow cytometry analysis (*n* = 3–4; *** *p* < 0.001; One-way ANOVA followed by Dunnett’s test). Data are presented as mean ± SEM

To establish whether IRDye-A20 is subjected to cellular internalization, incubations were carried out at 4 °C to inhibit active transport (Fig. 3A). Compared to 37 °C, incubation at 4 °C completely inhibited IRDye-A20 accumulation (*p* < 0.001). At 37 °C, MFI ranged from 12 700 to 33 800 a.u. in the concentrations range 10–500 nM, while MFI never exceeded 4400 a.u. at 4 °C. Thus, IRDye-A20 was internalized by active transport through αVβ6 integrin-mediated endocytosis. For IRDye-Scr, significant inhibition of internalization at 4 °C was observed only at the highest concentrations as 250 nM (*p* < 0.05) and 500 nM (*p* < 0.001), suggesting that IRDye-Scr internalization at these concentrations is mediated by active but non-αVβ6 specific transport. It should be noted that MFI after incubation with IRDye-A20 at 4 °C remained higher than that with IRDye-Scr at 4 °C (*p* < 0.05), which could be attributed to the fraction of IRDye-A20 attached to αVβ6 integrins but not internalized.

We further examined the mechanisms of IRDye-A20 endocytosis using specific inhibitors as EIPA, genistein and chlorpromazine that target, respectively, macropinocytosis, caveolae and clathrin-dependent endocytosis (Fig. 3B). Cellular uptake of IRDye-A20 and was not affected by EIPA and chlorpromazine treatment but was significantly reduced (by 30%) after pre-incubation with genistein (*p* < 0.001). These results suggest the predominance of caveolae-dependent mechanism of endocytosis.

### IRDye-A20 targeting in 3D cells

#### Homotypic-and-heterotypic-spheroids characterization

To establish 3D homotypic spheroids, HSC-3 tongue cancer spheroids (H10) were inoculated in a 96-well plate coated with 1% agarose, while stroma-rich cocultured spheroids (H5M5) were generated by seeding HSC-3 cells for 3 days, followed by the addition of MRC-5 healthy fibroblasts.

**Fig. 4:**
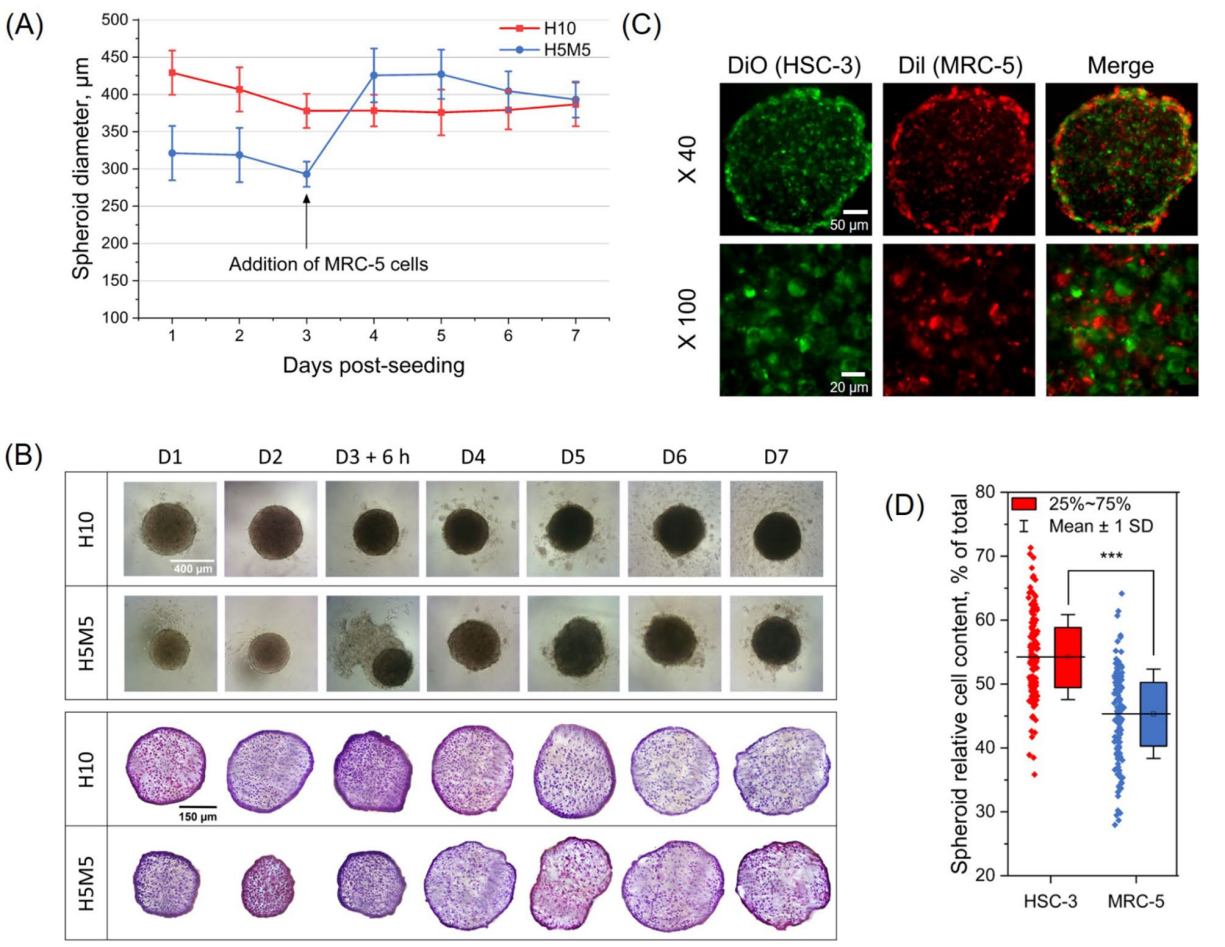
Multicellular tumor spheroid characterization. (**A**) Diameters of monocultured H10 and heterocultured H5M5 spheroids were measured until seven-day post seeding (*n* = 8–24). (**B**) Morphological monitoring of spheroids by inverted optical microscope (scale bar = 400 μm) and cryosection images of H10 and H5M5 spheroids stained with HE (scale bar = 150 μm). (**C**) Typical fluorescence images of cryosections of H5M5 spheroids at day 4 post-seeding. HSC-3 cells (green) were labeled with 2 µg/mL of PLGA-DiO and MRC-5 cells (red) were labeled with 2 µg/mL of PLGA-Dil (scale bar = 50 μm at x40 and 20 μm at x100). (**D**) Quantification of cellular subtypes in H5M5 heterospheroids by flow cytometry at day 4 post-seeding (*n* = 139; *** *p* < 0.001; Two Sample t test). Data are presented as mean ± SD

Spheroids diameter and its morphology were monitored until day 7 by inverted optical microscope (Fig. 4A). After three days culturing, H10 spheroids were about 375 μm in diameter and remained constant over the week. For cocultured heterotypic spheroids (H5M5) the diameter was ca. 300 μm until addition of MRC-5 cells and after that it gradually increased reaching 400 μm at day 7.

Morphology of spheroids was assessed by bright-field microscopy (Fig. 4B, upper panel) and displayed fully formed round spheroids H10 and H5M5. From day 5, in both types of spheroids, cells started to detach, and phenomena accentuated at day 7. HE staining (Fig. 4B, lower panel) demonstrated a decrease in nuclear hematoxylin pattern thus confirming a loss of cell density from day 4. Therefore, all further experiments with both types of spheroids were performed at day 4–5 after cells seeding. To assess cell repartition in H5M5 spheroids, each cell type was labeled with different fluorescent dyes before coculturing and cryosections were further assessed by fluorescent microscopy (Fig. 4C). DiI-prestained fibroblasts fully migrated into the spheroids and were uniformly present, from the spheroid boundary to the center. Relative content of cells subtypes in heterospheroids was examined by flow cytometry and revealed the presence of 55% of HSC-3 cells and 45% of fibroblasts (*p* < 0.001) (Fig. 4D).

### IHC and immunofluorescence characterization

**Fig. 5:**
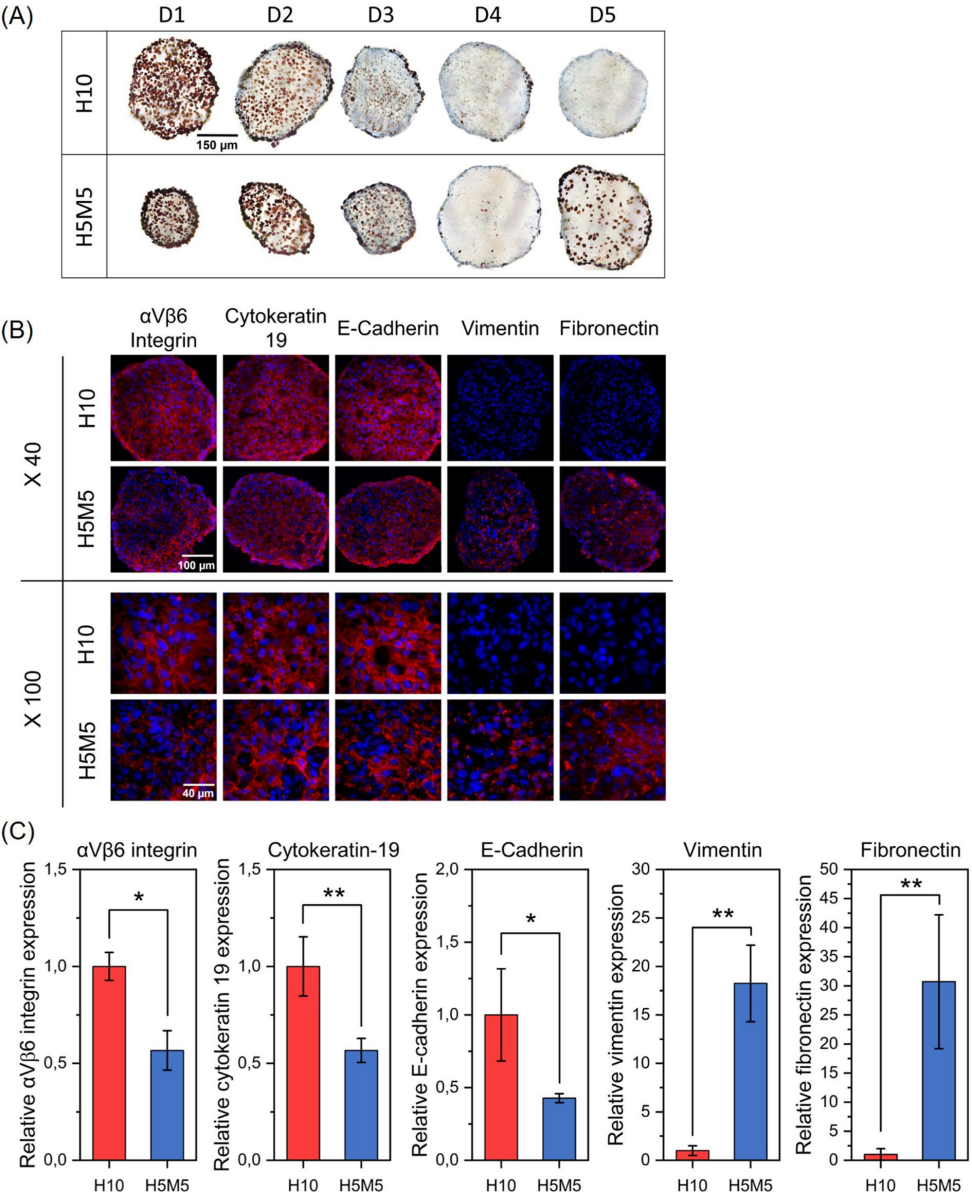
Multicellular tumor spheroid characterization. (**A**) Typical cryosection images of H10 and H5M5 spheroids after KI-67 immunohistochemistry staining (scale bar = 150 μm). (**B**) Typical immunofluorescence images of H10 and H5M5 spheroids cryosections stained with antibodies against αVβ6 integrin, cytokeratin 19, E-cadherin, vimentin, and fibronectin at day 4 post-seeding. Proteins of interest are in red and nuclei are in bleu after counterstaining with Vectashield-DAPI (scale bar = 100 μm at x40 and 40 μm at x100). (**C**) Relative quantification of immunofluorescence markers expression using ImageJ software (*n* = 3–4; * *p* < 0.05, ** *p* < 0.01; Two sample t test). Data are presented as mean ± SEM

Proliferation activity of both types of spheroids was characterized by KI-67 IHC staining (Fig. 5A). A high proportion of proliferative cells was found in homotypic spheroids at day 1, which progressively decreased with time with only few proliferative cells at day 4. Notably, addition of fibroblasts to H5 spheroids did not markedly improve KI-67 expression, suggesting that at least 24 h after their addition fibroblasts were not in the proliferative state. A notable difference was noted at day 5. While H10 spheroids remained unproliferative, proliferative cells were observed in H5M5 coculture.

Spheroid frozen sections were then subjected to immunofluorescence analysis to investigate αVβ6 integrin, cytokeratin-19, E-cadherin, vimentin, and fibronectin expression (Fig. 5B). Quantitative aspect of the expression of immunofluorescence markers was assessed by ImageJ analysis (Fig. 5C). In accordance with previous RT-qPCR and WB results in 2D cells, a strong and uniform αVβ6 expression was found in H10 homospheroids, while H5M5 heterospheroids were characterized by numerous negative blue (DAPI only) spots, corresponding to MRC-5 fibroblasts. Relative expression of αVβ6 (Fig. 5C) confirms immunofluorescent pattern.

Cytokeratin 19, an intermediate filament expressed by HSC-3 cancer cells, was uniformly expressed in H10 spheroids, but not in H5M5 spheroids after 24 h co-culturing (Fig. 5B-C). Conversely, vimentin, an intermediate filament characteristic of mesenchymal cells was clearly visible in H5M5 spheroids and was not expressed in H10 spheroids. Thus, both markers, cytokeratin 19 and vimentin confirmed the presence of two cell populations within coculture. Several studies have noted that the presence of junction proteins such as E-cadherin, are necessary for spheroid formation [48, 49]. E-cadherin was strongly expressed in H10 spheroids, allowing close interactions between HSC-3 cells. In coculture, E-cadherin expression was two times lower, probably restricted to HSC-3 cells. Fibronectin is known to be enriched in the tumor microenvironment and is one of the major pro-invasive ECM proteins. Fibronectin production is tightly related to the fibroblasts associated to tumor and as anticipated fibronectin was expressed only in fibroblasts-enriched spheroids.

### Accumulation of IRDye-A20 in spheroids

In the last step we evaluated IRDye-A20 potential as imaging agent on tumor spheroids and on stroma enriched tumor spheroids.

**Fig. 6:**
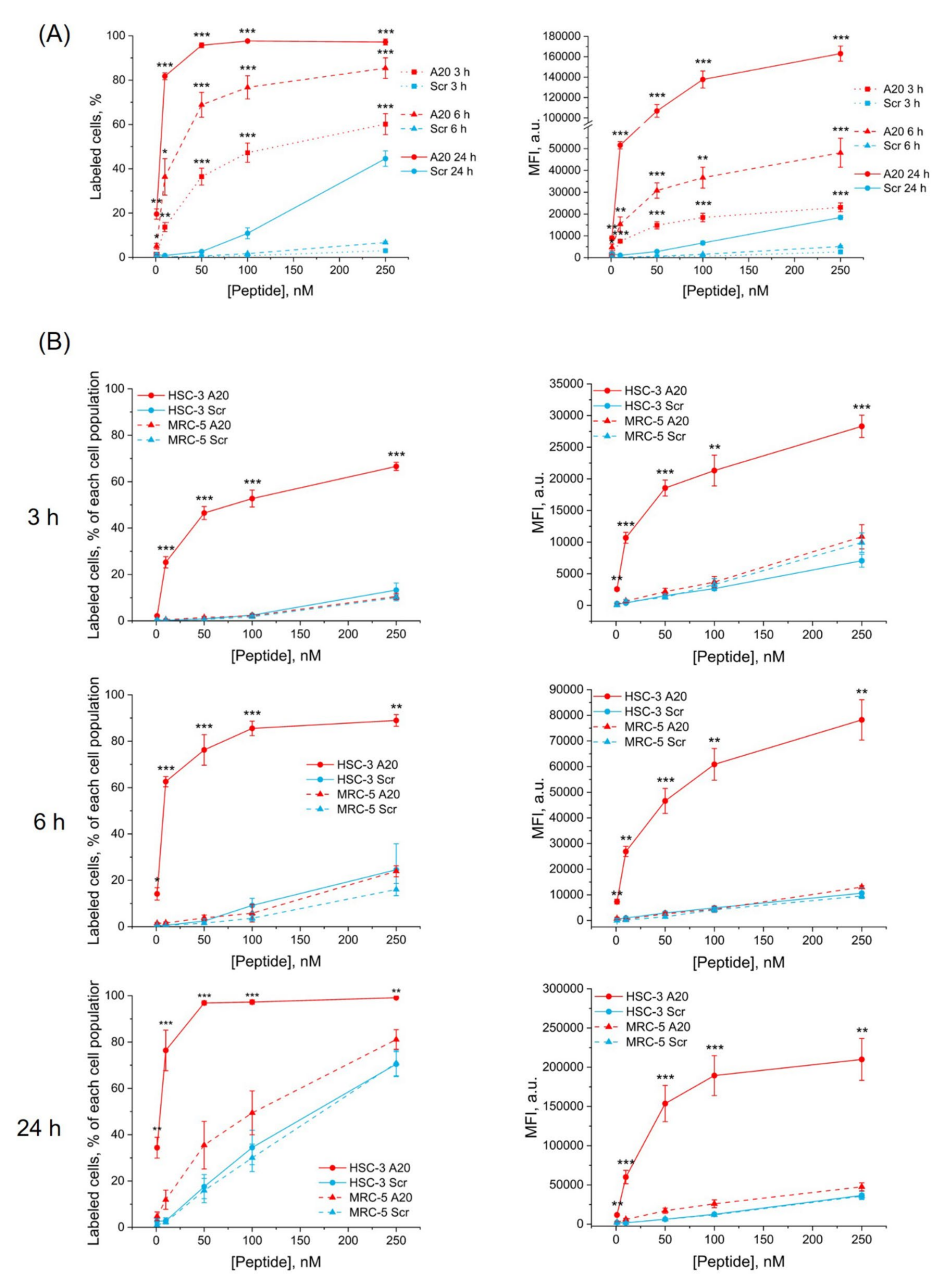
IRDye-A20 accumulation in spheroids. Flow cytometry analysis of H10 (**A**) and H5M5 (**B**) spheroids after incubation with IRDye-A20 or IRDye-Scr in the concentrations range 1-250 nM for 3-6-24 h. (**A**) Percentage (%) of labeled cells and MFI in H10 spheroid (*n* = 4–5; * *p* < 0.05; ** *p* < 0.01; *** *p* < 0.001; Two sample t test with Welch correction). (**B**) Percentage (%) of labeled cells and MFI in H5M5 spheroids (*n* = 5–11; * *p* < 0.05; ** *p* < 0.01; *** *p* < 0.001; One-way Welch Anova followed by Dunnett’s T3). Data are presented as mean ± SEM

H10 tongue cancer spheroids were incubated with IRDye-A20 or IRDye-Scr for 3 h, 6 h and 24 h before trypsinization and subsequent flow cytometry analysis (Fig. 6A). Two parameters were considered, namely the percentage (%) of labeled cells (left panel) and MFI (right panel). Incubation with IRDye-A20 from already 10 nM enabled higher labeling of H10 spheroids compared with IRDye-Scr at all incubation times (Fig. 6A). After 3 h, IRDye-A20 labeled up to 60% of spheroids cells against 3% with IRDye-Scr. After 6 h, cells labelling increased up to 85% with IRDye-A20 against 7% with IRDye-Scr. The profile of cell labeling was slightly different after 24 h incubation. At already 1 nM IRDye-A20 stained 20% of cells and nearly complete labeling (96%) was reached at 50 nM, while IRDye-Scr at 50 nM did not label more than 3% of spheroid cells. Concentrations of IRDye-Scr above 50 nM induced non-specific accumulation resulting in an increase of labeling from 11% at 100 nM to 45% at 250 nM after 24 h incubation. Free IRDye 680 accumulated slightly more than IRDye-Scr, but less than IRDye-A20, at both 3 h and 6 h incubation, while at 24 h incubation, accumulation was similar to that of IRDye-Scr (Supplementary Fig. S4).

If we consider the MFI parameter, at all used concentrations and all incubation times MFI with targeted IRDye was higher than that with IRDye-Scr. For example, MFI was 18 400 ± 1900, 36 700 ± 4800 and 137 700 ± 8400 a.u. at respectively 3 h, 6 h and 24 h incubation with 100 nM of IRDye-A20, while it reached only 6800 ± 850 a.u. after 24 h with 100 nM of IRDye-Scr (20-fold less compared to IRDye-A20).

To better recapitulate the impact of tumor microenvironment in oral cancers, we further addressed IRDye-A20 accumulation in fibroblasts-enriched tumor spheroids.

The number of labeled cells in H5M5 spheroids was evaluated at 3 h, 6 h and 24 h after incubation with IRDye-A20 or IRDye-Scr (Fig. 6B, left panel). Each cell population that makes up spheroids was analyzed separately to determine the percentage of labeled cells in HSC-3 and MRC-5 populations (Supplementary Fig. S5). IRDye-A20 cell labeling was always higher in HSC-3 cells compared to that in MRC-5 (Fig. 6B, left panel). Conversely, no statistically significant difference was observed in MRC-5 cells between IRDye-A20 and IRDye-Scr, consistent with an absence of αVβ6 expression in fibroblasts. At 3 h post-incubation, 46% of HSC-3 cells were labeled with 50 nM of IRDye-A20, against 1% labelling of MRC-5 cells. At 6 h, peptide labelling increased to 76% for HSC-3 and only to 4% for MRC-5. Finally, at 24 h, 97% of HSC-3 cells were labeled against 35% for MRC-5. Thus, with targeted IRDye we could achieve its selective accumulation in HSC-3 tumor cells.

Qualitatively similar results were obtained when we evaluated selectivity, applying MFI parameter (Fig. 6B, right panel). After 3 h incubation with 50 nM of IRDye-A20, MFI was 18 500 ± 1200 a.u. in HSC-3 cells and only 2200 ± 500 a.u. in MRC-5 cells. After 24 h, MFI increased up to 153 000 ± 23 000 a.u. in HSC-3 cells, and up to 17 000 ± 3300 a.u. in MRC-5 cells.

### Penetration of IRDye-A20 in spheroids

**Fig. 7:**
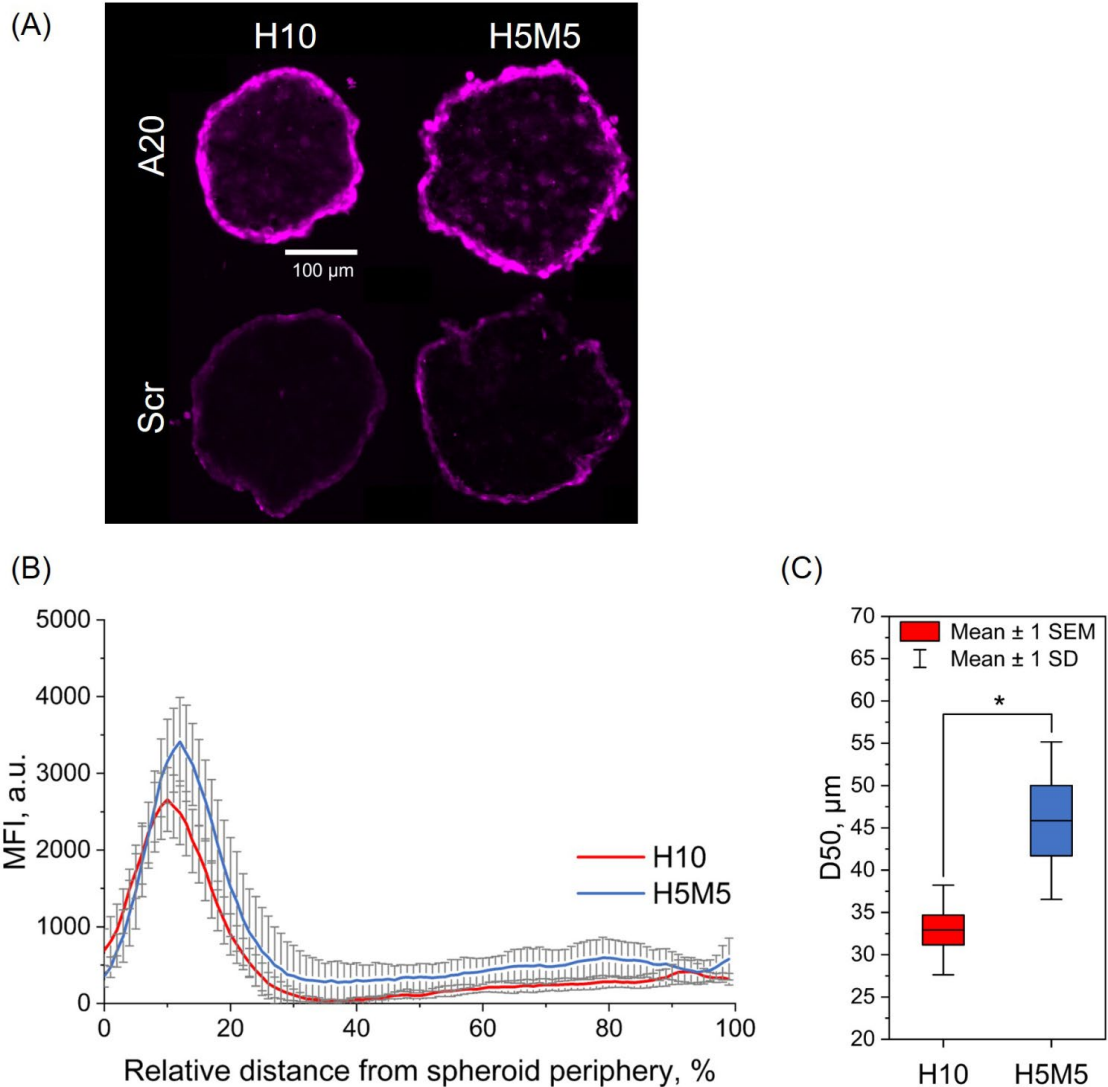
IRDye-A20 penetration in spheroids. (**A**) Typical fluorescence microscopy images of H10 and H5M5 spheroids cryosections after incubation with 250 nM of IRDye-A20 or IRDye-Scr for 24 h (scale bar = 100 μm). (**B**) Penetration profiles of IRDye-A20 (250 nM) in H10 and H5M5 spheroids after 24 h incubation. Data were obtained using ImageJ software by drawing 100 concentric circles and measuring MFI for each circle (*n* = 5–9). On the X-axis, 0% stands for spheroid’s periphery and 100% stands for its core. (**C**) Histogram, presenting D_50_ in H10 and H5M5 spheroids after 24 h incubation with IRDye-A20. D_50_ stands for the distance at which was reached 50% of maximum MFI. D_50_ were derived from panel (**B**) by calculating the cumulative MFI and determining the distance in spheroid corresponding to 50% of the maximum MFI (*n* = 5–9; *p* < 0.05; Two Sample t test). Data are presented as mean ± SEM

Finally, H10 and H5M5 spheroids were incubated for 24 h with IRDye-A20 or IRDye-Scr 250 nM for fluorescence imaging purposes. As illustrated in Fig. 7A, spheroids incubated with IRDye-A20 were much brighter than those incubated with IRDye-Scr. Unlike IRDye-Scr, IRDye-A20 fluorescence was observed across spheroids with a strong preference to the peripheral localization. Based on these images, MFI within H10 and H5M5 spheroids was quantified using ImageJ software (Fig. 7B). The distribution profiles of IRDye-A20 were similar for both spheroid models, however MFI was higher in H5M5 spheroid (peak at 3412 a.u.) compared to H10 spheroids (peak at 2658 a.u.). Moreover, the distribution profile was slightly shifted to the right in H5M5 spheroids, indicating increased penetration of IRDye-A20. In both cases, the majority of signal was found in the first quarter (25%) of the distance from spheroid periphery. Finally, we calculated the distance corresponding to 50% of the cumulative MFI (D_50_) (Fig. 7C). In H10 spheroids, the mean D_50_ was 33 ± 5 μm depth, while it increased up to 46 ± 9 μm in H5M5 spheroids (*p* < 0.05).

## Discussion

In the present work, we demonstrated the potential of IRDye 680 conjugated to A20FMDV2 peptide as a NIR imaging agent for OSCC spheroids. IRDye-A20 efficiently labeled all cancer cells in cocultured spheroids through αVβ6 mediated internalization while leaving unlabeled healthy fibroblasts. Therefore, we achieved a contrasting labeling with IRDye 680CW conjugated to A20 peptide.

We have developed a homotypic spheroid model composed of tongue cancer cells HSC-3 and heterotypic 3D spheroids of stroma-enriched HSC-3 cells. This was achieved by adding MRC-5 fibroblasts to tumor HSC-3 spheroids. Both mono and cocultured spheroids were compact, round shaped and had relatively constant diameter over the week. Fibroblasts, initially surrounding the spheroid, completely penetrated it within 24 h of coculturing. Proliferative state of cells analyzed by KI-67 IHC revealed a time-dependent reduction of cell proliferation 4 days after cell seeding. However, 48 h after addition of fibroblasts, we observed a re-start of cell proliferation. Spheroids were further characterized by immunofluorescence, which confirmed the presence of two distinct cell populations in coculture and revealed an enriched extracellular matrix, evidenced by fibronectin expression. Such ECM is of major interest in assessing the therapeutic potential of various molecules since ECM affects the behavior of cancer cells, promoting cancer progression [50], and can act as a barrier to therapeutics penetration across the tumor [51].

To target tongue cancer cells for imaging purposes, we used the NIR fluorescent contrast agent IRDye. Indeed, molecules of the IRDye family (IRDye 680, IRDye 750, IRDye 800) have been the subject of numerous fluorescence guided surgery investigations in preclinical models as well as in clinical trials [52, 53]. This widespread use of NIR fluorescence is related to the deep red/infrared light penetration and reduced tissue autofluorescence [54–56]. Indeed, as was reported for human maxillary sinuses mucous tissue, IRDye 680 provides penetration thickness of 3.6 mm [57] and this penetration depth largely exceeds the thickness of mucous membrane both in normal and in pathological tissues. Cell targeting was achieved by conjugating IRDye 680 with the A20FMDV2 peptide, known for its very high binding affinity to αVβ6 integrin [26, 27]. Consistent with other observations [47] we found an overexpression of αVβ6 in 2D and 3D tongue HSC-3 cancer cells while no expression was found in healthy MRC-5 cells. Our RT-qPCR and western blot analysis corroborate with ITGB6 expression in primary tumor samples and its normal counterparts through UALCAN analysis, demonstrating a significant overexpression of this protein in tumor samples compared to controls.

We evaluated the targeting abilities of IRDye-A20 in both HSC-3 (αVβ6+) and MRC-5 (αVβ6-) 2D monolayers and demonstrated a specific labeling of HSC-3 cells with IRDye-A20 in the 10–100 nM range. Conversely, no targeting was observed for IRDye-A20 or IRDye-Scr in MRC-5 cells, confirming the need of αVβ6 integrin to mediate product accumulation. It is important to note that incubation of IRDye-conjugated peptide in serum-free medium for 24 h could induce cells starvation potentially altering drug delivery mechanism as was already reported for other peptides–targeting medicines [58]. We performed 24 h incubation of IRDye-A20 in serum-free and serum-rich medium in 2D cells. Our results showed that accumulation of IRDyeA20 was twice higher when incubated in the presence of FBS compared with serum-free medium (data not shown).

Further, active transport was inhibited by incubating cells at 4 °C to establish whether IRDye-A20 was bound to cell membrane through αVβ6 integrin or it was rather internalized. Results demonstrated that IRDye-A20 was internalized confirming active transport of IRDye-A20. Using different endocytosis inhibitors, we showed that IRDye-A20 was endocytosed through caveole-dependent pathway. Similar findings were observed in Meecham et al. study, which concluded on caveole- and clathrin-dependent endocytosis of the ligand bound to αVβ6 integrin with a strong predominance of the caveole-dependent pathway [45].

After demonstrating the effectiveness of IRDye-A20 in monolayers, assays evolved towards monoculture spheroid model H10 to better mimic the issues and challenges related to tumor targeting such as the barrier effect, the stratification of cells into different layers (proliferative, quiescent, necrotic), the effect of gradients (oxygen, nutrients, wastes, pH) [59]. IRDye-A20 labeled H10 spheroids cells in a time and concentration-dependent manner, labeling the whole spheroid after 24 h with a preferential accumulation on the periphery of spheroid. In the meantime, IRDye-Scr was ineffective, labelling about 3% of cells. We further carried out the study on the stroma-rich coculture 3D H5M5 model. Analysis was performed separately in each cell population (tumor cells vs. fibroblasts) by discriminating them by pre-labeling of HSC-3 cells with PLGA-DiO NPs. IRDye-A20 labeled HSC-3 cancer cells but not MRC-5 fibroblasts, confirming targeting selectivity in 3D cocultured models. Moreover, MFI of cancer cells was much higher than those of fibroblasts after 24 h incubation with IRDye-A20. Similar results were obtained comparing HSC-3 labeling in monoculture H10 and in coculture H5M5. Thereby, the presence of stroma did not interfere with cancer cells labeling. Considering MFI, its increase in cocultured spheroids may be attributed to physical factors such as spheroid loosening. In fact, E-cadherin, a junctional protein important for spheroid formation [48, 49], is not expressed by fibroblasts. Thus, intercellular contacts may be less cohesive in the H5M5 coculture model, in contrast to the H10 model where all HSC-3 cells express E-cadherin, thus improving IRDye-A20 penetration.

Currently, the development of FGS in HNSCC focuses mainly on two approaches: passive targeting with indocyanine green (ICG) [10, 11] or active targeting through anti-EGFR antibodies such as cetuximab and panitumumab [12, 13, 15, 16]. However, tumor labeling with ICG does not always offer a significant contrast between healthy and cancerous tissue. Concerning EGFR targeting, its contribution to FGS has been demonstrated in numerous studies. Here, the advantage of targeting αVβ6 integrin is that unlike EGFR, it is not expressed in healthy cells, thus providing better imaging contrast [22]. The approach based on αVβ6 integrin is recent and has been poorly explored in HNSCCs. Furthermore, a targeting approach based on peptide has the advantage of enabling production on a larger scale and at a lower cost once the therapy has been approved in clinical settings [60]. However, improvements of the molecule formulation, particularly by pegylation, will be necessary to envisage in vivo studies,

## Conclusion

To conclude, the present work demonstrated an efficient selective targeting of A20FMDV2-conjugated IRDye 680 in 3D stroma-enriched tumor environment. Considering that αVβ6 is overexpressed at invasive margins and that the majority of oral tumors are in advanced stage at detection, a high potential of A20FMDV2 coupled to infrared tracer should not be underestimated. Indeed, a cutoff of 1 mm or less between cut tissue edge and invasive tumor is considered as a close margin at the risk of recurrence [61]. As was demonstrated by the Monte Carlo radiative transport simulations, the wavelengths 680–700 nm penetrates up to 1.3 mm with 50% of the intensity reaching this depth [62].

Optimization of the compound formulation with a successive in vivo assay will provide a better imaging potential of this IRDye-A20 molecule. The addition of PEG residues to IRDye-A20 could be an efficient way to improve dye accumulation as was already demonstrated in vitro in melanoma and pancreatic cancer cells as well as in vivo on melanoma-xenografted mice [31, 63].

### Electronic supplementary material

Supplementary Material 1

Supplementary Material 2

Supplementary Material 3

Supplementary Material 4

Supplementary Material 5

## Data Availability

The datasets used and analyzed during the current study are available from the corresponding author upon reasonable request. Clinical data used in this article can be found athttps://ualcan.path.uab.edu

## Abbreviations

A20: A20FMDV2
CAR T-cells: Chimeric antigen receptor T-cells
DMEM: Dulbecco’s Modified Eagle Medium
ECM: Extracellular matrix
EGFR: Epidermal growth factor receptor
FBS: Fetal bovine serum
FGS: Fluorescence guided surgery
HE: Hematoxylin and eosin
ICG: Indocyanine green
IF: Immunofluorescence
IHC: Immunohistochemistry
ITGB6: integrin β6 subunit
MEM: Minimum Essential Medium
MFI: Mean fluorescence intensity
NIR: Near Infrared Fluorescence
OSCC: Oral squamous cell carcinoma
PLGA: Poly(lactic-co-glycolic acid)
PMSF: Phenylmethylsulfonyl fluoride
QoL: Quality of life
Scr: Scramble
TFM: Tissue freezing medium
