# Supplementary figures and images for: Targeting of 3D oral cancer spheroids by αVβ6 integrin using near-infrared peptide-conjugated IRDye 680
Source: Cancer Cell Int. 2024 Jun 29;24:228. doi: 10.1186/s12935-024-03417-y (PMC11218202; doi:10.1186/s12935-024-03417-y)

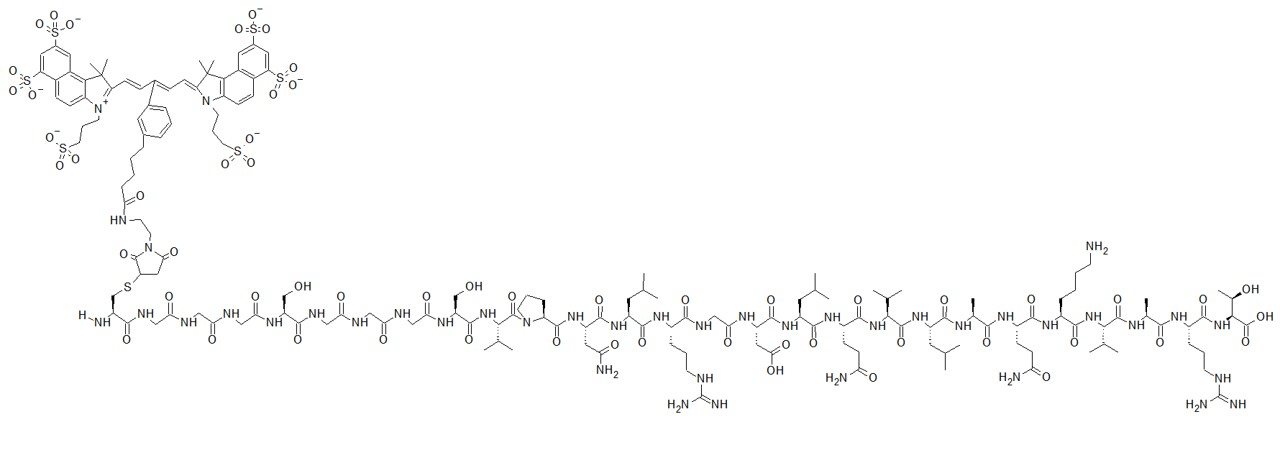

Supplement: Supplementary file 1 — Supplementary Material 1 [file 12935_2024_3417_MOESM1_ESM.jpg]

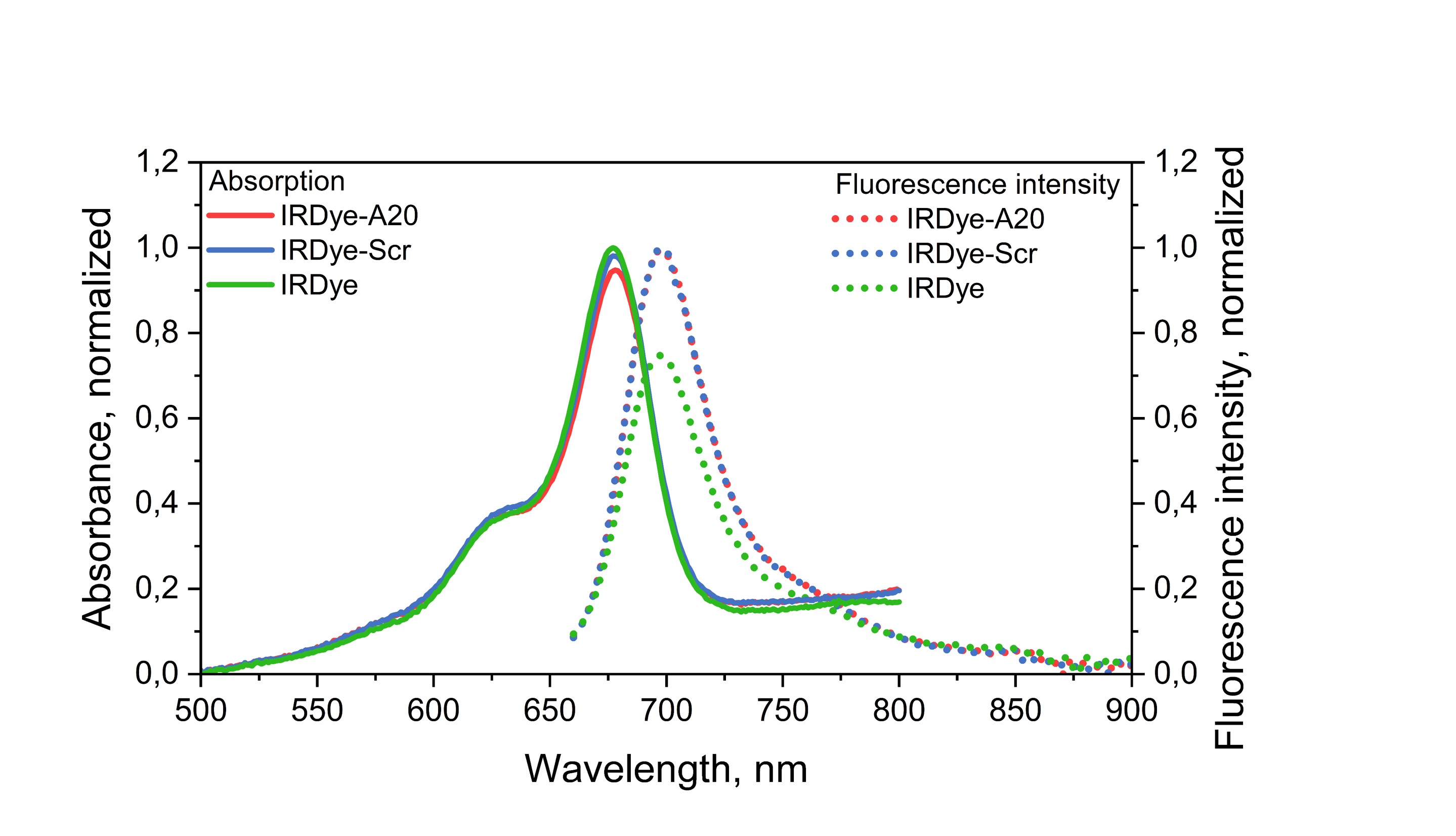

Supplement: Supplementary file 2 — Supplementary Material 2 [file 12935_2024_3417_MOESM2_ESM.jpg]

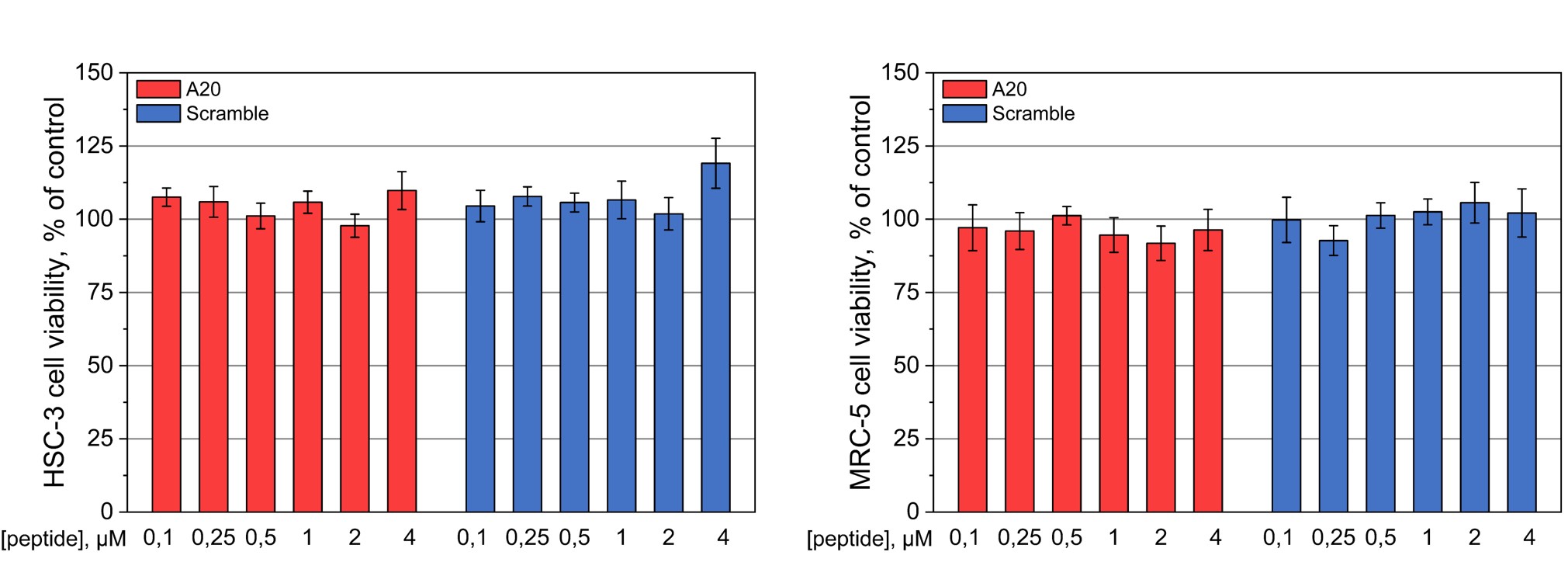

Supplement: Supplementary file 3 — Supplementary Material 3 [file 12935_2024_3417_MOESM3_ESM.jpg]

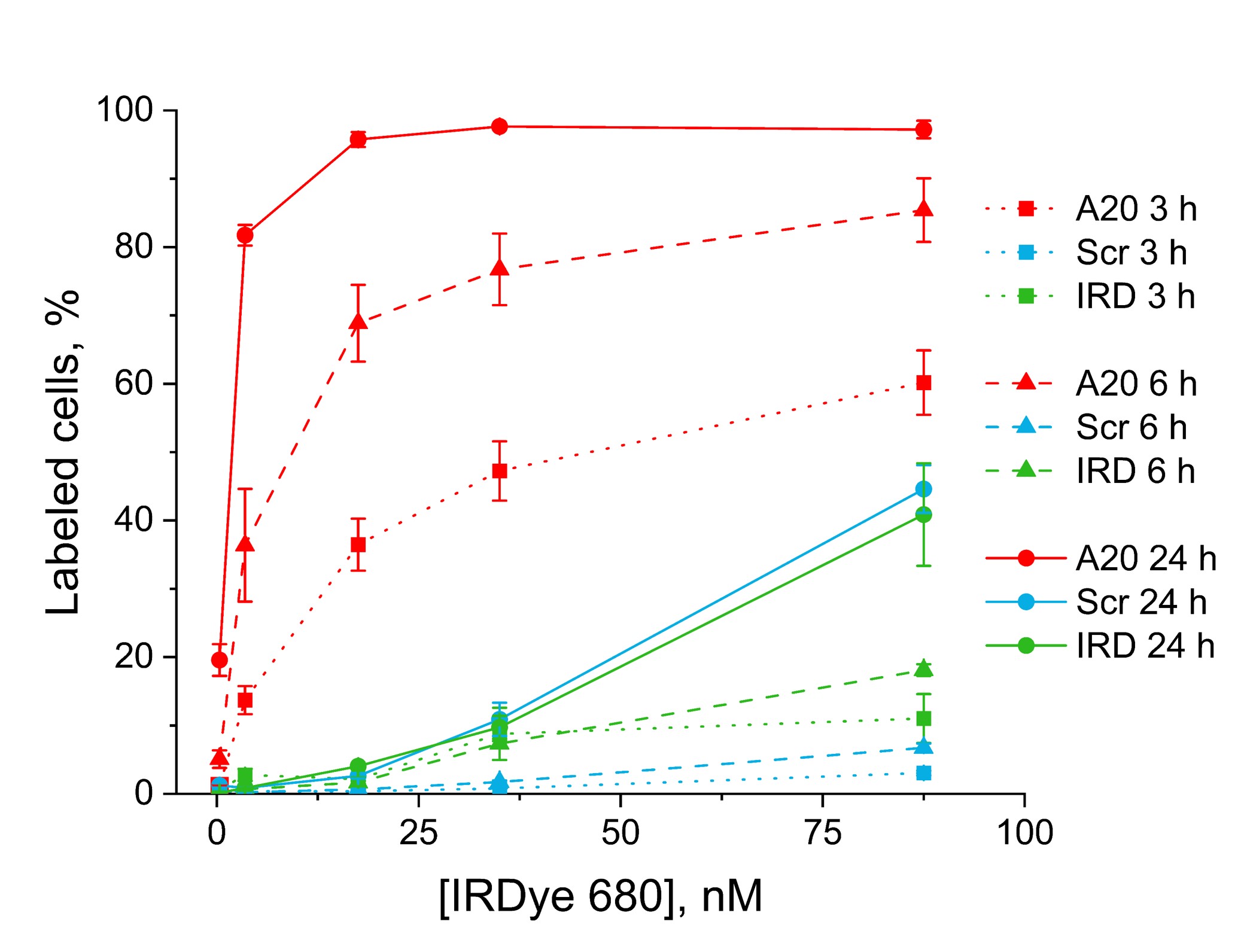

Supplement: Supplementary file 4 — Supplementary Material 4 [file 12935_2024_3417_MOESM4_ESM.jpg]

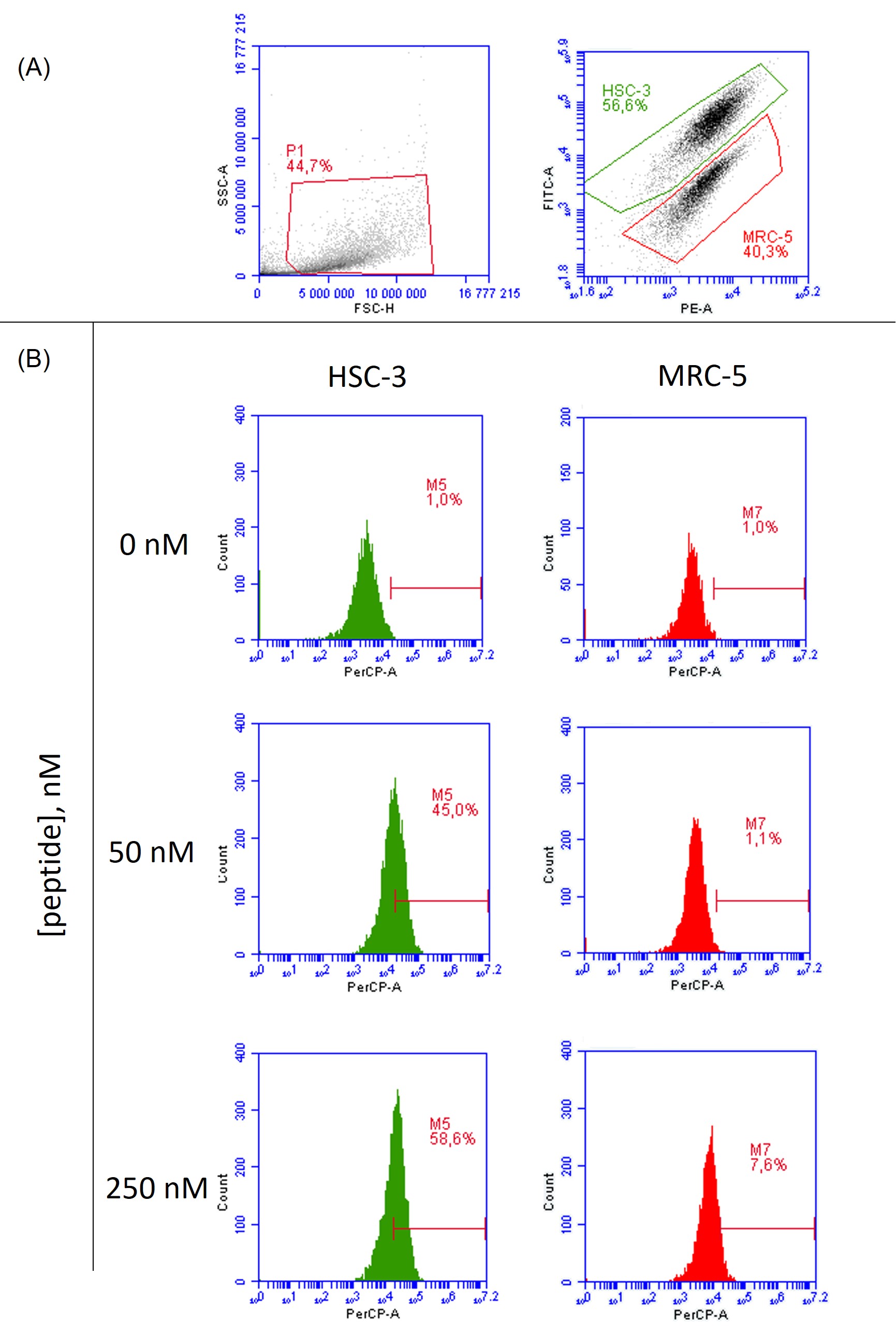

Supplement: Supplementary file 5 — Supplementary Material 5 [file 12935_2024_3417_MOESM5_ESM.jpg]
